# The Climate of the United States and Its Endemic Influences

**Published:** 1843-02

**Authors:** 


ART II.—*The Climate of the United States and its Endemic Influences. Based on the Records of the Medical Department and Adjutant General's Office, United States Army.* By SAMUEL FORRY, M. D.

*L' ensemble de toutes les circonstances naturelles et physiques, au milieu desquelles nous vivons dans chaque lieu.*—CABANIS.

*The best observations upon climate often lose half their value for the want of an exact description of the surface of the country.*—MALTE-BRUN.

*New York: J. & H. G. Langley,* 57 *Chatham street; Barrington and Haswell, Philadelphia; Little and Brown, Boston,* 1842; p. 378 12mo.

In respect to works of its own class, this volume may be regarded as THE BOOK OF BOOKS—or it *might* be called *the* book—there being, as far as we are informed, no production resembling it, either in the English, or any other, language. Assuredly there is none *strongly* resembling it; much less is there any one *equal* to it, in many of its most interesting and important qualities. It is therefore not only *original*, but *unique* in its kind.

To characterize it still further, it is the first work written on the subject of general American medical statistics, and is therefore, in its object entirely new. Its basis is spacious, being co-extensive with the outline of the United States; its matter consists of facts of great value, collected by observation continued, with uncommon labor, perseverance, and pains-taking, through a period of *twenty years;* these facts are composed of the manifold and important elements that enter into the composition of the different climates of the United States, and the effects, salutary and deleterious, which those climates produce on organized matter; and of the changes which the climates have already sustained, since the settlement of the country, and are still sustaining, by the action of certain specified causes. And to all this is added much information, curious as well as useful, respecting the changes and their effects, that have occurred in the climates of other countries, within the period of historical records.

Nor, are these facts, interesting and valuable as they are, all that confers on the volume its title to the high standing it is destined to attain. Far from it. The excellence of its plan and arrangement, the elevation and even grandeur of its aim, the masculine spirit and love of philosophy which pervade it, and the elegant style in which it is composed, are in no respect inferior to the matter which it contains.

Should any one competent to sit in judgment on this production suspect us of bestowing, in these expressions, undue commendation on it, let him attentively study it, and he will be made sensible of his mistake. We say, "study it;" for the whole amount of its worth is not to be fully apprehended and weighed by a common-place perusal of it.

But we have not yet mentioned the special purposes which our author had in view in the preparation of the work. That our readers therefore may have a knowledge of them the more exact and definite, we shall state them in the writer's own words.

"The chief objects intended to be accomplished by the publication, are to present, in Part First, a classification of the principal phenomena of our climate, *physically* considered; and to attempt in Part Second, to trace out the *medical*

relation of these laws, thus establishing in both a classification of climates having for its basis observation."

In more detailed, and perhaps more communicative language, the design of our author is, to point out to what extent, and by what agents, climate (including, as two of its elements, special and influential localities, and given geological formations) is instrumental in the production of such complaints as prevail epidemically in different situations. And it will not be denied, that the object thus held in view by him constitutes one of the weightiest and most interesting desiderata connected with the medical literature of our country. And the ability, with which he has executed his design, places, in that respect, the medical literature of the United States above that of any or all of the nations of Europe. For, as already mentioned, we have yet to learn that a single work of the kind has heretofore appeared, in any language, comparable to that which now lies before us. And we are gratified to learn that, in this sentiment, some of the European Journals fully concur, and, with that liberality and magnanimity, which should always characterize scientific productions, avow that concurrence.

We know of only three persons who have heretofore made the climate of the United States a subject of discussion to such extent as is worthy of notice. But they were men of great knowledge, ability, and distinction—Mr. Jefferson, Dr. Rush, and Mr. Volney. For want of an acquaintance however with certain branches of knowledge indispensable to their purpose, neither of them was qualified for the task he undertook. The reason is plain. Neither of them was versed in either meteorology or geology, and, of the three, Dr. Rush alone was acquainted with medicine. And, as a qualification for a *thorough discussion* of the subject of climate (such discussion we mean as that we are now noticing) a knowledge of those three branches of science is essential. And Dr. Forry has shown that his qualifications in those and all other respects, are amply sufficient for the accomplishment of the enterprise in which he engaged.

With such qualifications, united to the persevering labor he bestowed on the subject, the result corresponds. Not only has he shown himself to be far superior to his three distinguished predecessors in the *amount*, but also in the *accuracy*, of his knowledge of the climate of the United States. Hence he has satisfactorily corrected sundry errors which they had committed; and by which, through the influence of their writings, and the weight of their names, the public mind has long been misled. Dr. Forry moreover has manifested in his treatise as much of sound judgment and practical common sense, as he has of an acquaintance with letters, and with the principles of the several branches of science, to the study of which he has so successfully devoted himself. If we mistake not, such will be the standing and effect of the work from his pen, which we are now considering, that it will be instrumental in producing, at least among his countrymen, if not on a more extensive scale, the commencement of a new era in the cultivation of climatology. Our author's definition of climate alone is indicative of his entire mastery of the subject. He has given a much more thorough and comprehensive exposition of it as an aggregate, as well as a more full and accurate detail of the elements which compose it, than is to be found in any other work, with which we are acquainted. That our readers may examine and judge of it for themselves, and derive from it the information it is calculated to impart, we shall present them with the passage containing the definition:

"The term *climate*, which is limited, in its rigorous acceptation, to a mere geographical division, and in ordinary parlance, to the temperature only of a region, possesses in medical science, a wider signification. It embraces not only the temperature of the atmosphere, but all those modifications of it which produce a sensible effect on our organs, such as its serenity and humidity, changes of electric tension, variations of barometric pressure, the admixture of terrestrial emanations dissolved in its moisture, and its tranquility as respets cboth horizontal and vertical currents. Climate, in a word, as already defined, constitutes the aggregate of all the external physical circumstances appertaining to each locality in its relation to organic nature. "To observe," says Professor Rostan, "the simultaneous effects of light, heat, electricity, of the winds, &c., on the organic productions of the different zones of the earth, to explore the nature of this earth, to deduce from this knowledge the influence which they exercise on the physical and moral nature of man, such is the wide field which climates present to investigation."

We have said that our author's mind is judicious and practical, no less than scientific and literary. In illustration and proof of this, it is necessary for us only to say, that his application of climate, as just defined, to the promotion and preservation of health, the production and modification of disease, and of course to the furtherance of the public good, is in strict accordance with his views of its composition and nature, as an agent, exercising an influence on organized matter. Hence his division of the United States into three great compartments—the Northern, Middle and Southern—in each of which the diseases that prevail correspond to the predominant elements of the climate of the place, in full and fair correspondence with his representation of them. In further explanation of our meaning on this topic, we shall extract from his publication another passage.

"The connexion between meteorology and medical science is, in truth, highly important. From the days of Hippocrates, the records of medical philosophy demonstrate that the phenomena of life are not the result of original organization only; but that the moral, intellectual, and physical capacities of man are subject to the influences of those causes, the aggregate of which constitutes climate. The doctrine receives an apposite elucidation in the corporeal degeneration induced by malaria. So deep and pervading are the effects of this subtle poison on the indigenous inhabitants of marshy districts, in warm climates, that the energies of the system are sapped, and premature decrepitude induced; and when subjected to these baneful exhalations, through successive generations, the mind becomes torpid and imbecile, the moral sentiments debased, and the stature and symmetry of the body deteriorated. Again it finds a ready illustration in the history of the recent epidemic (*cholera asphyxia*) which, in its wide diffusion, threatened to depopulate vast tracts of the earth's surface; but which, owing doubtless to great meteorological changes, notwithstanding inappreciable by our eudiometric instruments, suddenly ceased its ravages, and left, like many other destructive pestilences in preceding ages, scarce a trace behind but the terror of its name."

It is perhaps due from us to our author that we say something more definite and characteristic, than we have yet done, of his style, to which we do no more than justice, when we pronounce it eminently spirited, graphic, and scholar-like. In one extract more it shall speak for itself; in doing which it will testify conclusively to the correctness of our remark.

"But woe to the invalid that braves the torments of a summer residence" (in a burning climate,) "under the disadvantage of a camp life! Insects are the pests of a tropical clime. As to fleas, flies, and ticks, the interior of Florida may well rival Egypt in the days of Pharoah. The chigoe (*pulex penetrans*) insinuates itself beneath the skin, where it soon establishes a populous colony. Flies seem indeed to form a component part of your food, your drink, and the atmosphere you inhale. Lizards, snakes, and scorpions get into your bed, whilst the industrious ant and weevil not only eat your rations, but devour your books—the food of the mind. All nature seems alive; and every hour you observe some uncouth living thing, whose family name has scarce been registered by the entomologist. In addition to these annoyances, the ear will be greeted by a nightly serenade performed by wolves and alligators—a woful concert of whining yells and dismal bellowings, constituting the realization of a *howling* wilderness."

Notwithstanding however the high estimation, in which we hold the work before us, *as a whole,* there are certain positions in it, in which our own observation, connected with reflection, forbids us to concur. We shall briefly state two of them, both of which relate to winds.

We believe and have long believed with Dr. Forry, that the Gulf-stream and the immense "ocean-lake" which it forms, expend much—perhaps most of their tepefying influence on the atmosphere of the northern and eastern Atlantic, and on that of the western shores of Europe and Africa. For that Africa as well as Europe feels that influence, cannot we think, be reasonably questioned.

We are strongly apprehensive, however, that the climate of the United States is more affected by the Gulf-stream, than the Doctor seems fully prepared to admit. This is the case, in particular, during the prevalence of a strong east, or southeast wind. The latter wind more especially throws, in few hours from its commencement, the whole mass of the Gulf-stream atmosphere on the shore of the States to which it is directed.

This wind, when it begins to blow, is comparatively *cool;* because it brings with it the temperature of the ocean-atmosphere between the Gulf-stream and the American coast. But in the space of from three to five hours, and sometimes less, it becomes warmer by several degrees; because it then possesses much of the temperature of the atmosphere of the Gulf-stream. We *might* say that it is then nothing but a current of that atmosphere setting toward the west. Thus is the climate of our eastern border rendered hotter by it in summer, and milder in winter. It is worthy of remark however, that, during the latter season, south-easters are rare with us.

The same wind moreover is often, if not usually, instrumental in the formation of clouds, which descend for the most part in torrents of rain. The reason of this is plain. The warm air comes loaded with humidity from the ocean, which, when sufficiently condensed, by the coolness of the land-air, assumes, by attraction, the shape of water-drops, and falls, by gravity, in the form of rain. So much for the south-east wind. Now for a few remarks on its antagonist.

The north-west wind is the coldest that is experienced in the United States. The reason of its low temperature, as Dr. Forry and most if not all other persons allege, is, that it comes from the cold atmosphere of what is usually called the "American Tartary," far beyond the region of the Lakes.

In this hypothesis we cannot concur; because we believe it groundless and untenable. In our brief discussion of it, we shall express ourselves as if resident in the city of Philadelphia, because we there first made it a subject of examination and thought. We therefore now consider ourselves remote from the frozen and snow-covered plains of the American Tartary, at least from fifteen hundred to two thousand miles—we believe much more.

No wind short of a hurricane travels *fifty miles* an hour. The north-west wind rarely travels more than from *twenty* to *thirty*. But we will give it the rate of *forty*, which we know to be not a little beyond its usual velocity. On the supposition that its starting point is distant from us two thousand miles, it will reach us in *fifty* hours—and if only fifteen hundred, in a few minutes more than thirty-seven hours.

Nearly that space of time then must elapse, after the commencement of the north-west wind, before it can reach us in Philadelphia, and cool materially the temperature of the atmosphere.

But is such the case? Does a period of that extent pass by, before the frigorific influence of the wind is experienced? Far from it. Not a tenth of the space expires before its effect is very sensibly, not to say severely felt.

We have witnessed a depression of the mercury, by the chilling action of the north-west wind, to the extent of *thirty degrees, in less than three hours.* We once at least, if not oftener, knew a change of the kind to be produced, in very little more than *one* hour.

From the region far north and west of the lakes then, it is absolutely impossible for that cold wind to come. We deem it highly questionable, whether a wind of such extent has ever existed. A stream of air, over mountains and hills, two thousand, or even fifteen hundred miles long, would be, we apprehend a new phenomenon. The areas of such atmospheric movements are usually limited. Mariners know that ships at sea but a day's sail from each other, or perhaps less, where there is nothing to obstruct the sweep of the air, often experience at the same moment very different weather While one is in a gale, its neighbor is becalmed. We once sailed from London, in company with an American ship, bound like ourselves to the United States. So great was our superiority in sailing-speed, that, in a few hours, we left our companion *out of sight* behind us. Yet *she* made her passage, under fine weather and fair winds, in about *twenty-five* days; while *we*, pursuing a track not very distant from hers, were detained on the water by heavy gales, head-winds, cross-winds, and no winds at all, *fifty-two* days. Nor are events of the kind unusual.

Whence then, it be may asked, does the north-west wind come? It appears and has long appeared to us to come from above, in such an earthward direction, as to bring down with it the higher and colder strata of the atmosphere, whose temperature is at, or perhaps below the freezing point, and mingle them with the lower and warmer strata, thus reducing the latter to a medium temperature.

Are we asked, why we believe that the north-west wind comes to us in a direction more perpendicular than that of other winds? We reply, that the phenomena attending it appear to us to testify strongly to that effect. It makes our chimnies smoke much more certainly and annoyingly than any other wind. It even rushes very often directly down the chimney, blowing soot, smoke, and ashes out of the fireplace. Nor is this all. It comes in puffs and flaws, which render it unusually dangerous to sail-boats. While other winds make their approach horizontally, producing a ripple on the water, which warns the boatman to be on his guard; it pounces on him from above, like an eagle on its prey, and not unfrequently capsizes his wherry. Previously to the construction of steam-vessels, three-fold more ferry-boats were upset, on the river Delaware, by the north-west wind, than by those from all other points of the compass.

The opinion here expressed respecting the source of the coldness of the north-west wind, we have entertained and defended almost from our boyhood; and we published it early in the present century, accompanied by the reasons which we deemed confirmatory of it. And though we do not pronounce it incontestable, yet, as far as we are informed, it has never *been* contested. One word more before we dismiss it.

M. Volney inserted the opinion in his treatise on the climate of the United States, and considered it well-founded and sound. In thus stating it, and sanctioning it by his name, he made no reference to us. Yet we solemnly declare, that he was *indebted* for it to us; though we were quite youthful at the time. Nor is this the only opinion that M. Volney *borrowed* from Americans, and inserted in his book, without acknowledgment. In truth, though extensively informed, he was *a plagiary*—and not *an original* either in observation or thought.

Are we again asked, why it is that the north-west wind should come to us, in a direction more perpendicular, than that of any other wind? We reply that we do not know. But our ignorance of the cause detracts in no degree from either the *possibility*, or the *probability* of the fact. We do not belong to that class of *wise ones*, who discredit facts, merely because they *do* not and cannot comprehend their causes. When we look thoroughly into the matter, facts are the only objects of our knowledge. Of causes, whether moral, intellectual, or physical, and of their mode of operating, we are perfectly ignorant. On the present subject analogy may throw some semblance of light. At least it may make it appear, that a wind pursuing a downward direction is neither an *unnatural* nor a *preternatural* phenomenon.

It is well known that down the sides of the Andes, the Alps, the Pyrenees, and other lofty and snow-covered mountains, streams of cold air frequently descend into the vallies at their feet, suddenly and greatly reducing the temperature of their atmospheres. Nor do these cold winds pass over the tops of mountains, and then sweep downward along their sides. They come from their lofty regions, but not from their tops. Yet we know not why the air thus descends. But we know the fact, and confide in its truth. And we further know, that when a wind passes *over the top* of an elevated mountain (which it sometimes does) it pursues a downward direction, along its opposite side. In truth there is no other direction which it can pursue. A downward wind therefore is not prohibited by the laws of nature. Nor can we render any reason why a stream of cold air may not descend through the atmosphere in other places, as well as down the inclined plain of a mountain.

Another phenomenon connected with our subject may be cited. Every tornado, violent thunder-storm, and other fierce commotion in the asmosphere, diminishes its temperature. And this effect it produces by mingling together the higher and lower strata of air.

Though these remarks do not solve our problem, respecting the perpendicular direction of the northwest wind, they have a tendency to render it more familiar to us—and also the more to persuade us perhaps of its *probability.* At any rate, no hypothesis can be devised more perfectly groundless—assuredly not more *improbable*, than that which transports the cold of a northwester to the coast of the Atlantic, and even hundreds of leagues along its surface, from the frosty atmosphere of a country far beyond the lakes.

But we must bring to a termination this notice, which, though longer than we designed to make it, has disclosed but a mere fraction of the value of the volume we have been considering. Of even the leading elements of it, to which it was our intention to refer, we have been able to embrace in our remarks but a few. In truth, as already intimated, the entire and abundant amount of that value can be compassed only by a thorough acquaintance with the production which exhibits it. As that production, however, merits, as highly as any other of American origin, instead of a mere notice like the present, a full, regular, and well prepared review, it may perhaps be treated by us somewhat to that effect in a future article. Meantime we earnestly recommend it to the attention and study of such readers, as covet at once high gratification and important instruction.

